# Utility of remission criteria for the renal prognosis of IgA nephropathy

**DOI:** 10.1007/s10157-021-02069-w

**Published:** 2021-05-11

**Authors:** Keiichi Matsuzaki, Hitoshi Suzuki, Tetsuya Kawamura, Yasuhiko Tomino, Yusuke Suzuki

**Affiliations:** 1 Department of Nephrology, Juntendo University Faculty of Medicine, Hongo 2-1-1, Bunkyo-Ku, Tokyo, 113-8421 Japan; 2 Division of Kidney and Hypertension, Department of Internal Medicine, The Jikei University School of Medicine, 3-25-8 Nishi-Shinbashi, Minato-Ku, Tokyo, 105-8461 Japan; 3 Progressive Renal Diseases Research, Research on Intractable Disease, from the Ministry of Health, Labour and Welfare of Japan, Tokyo, Japan

**Keywords:** IgA nephropathy, Renal prognosis, Remission criteria

## Abstract

**Background:**

Novel criteria for the remission of Immunoglobulin A nephropathy (IgAN) based on an opinion survey of Japanese nephrologists and literature review were proposed in 2013. This single-center, longitudinal retrospective cohort study was conducted to validate this criteria.

**Methods:**

Present study included the IgAN patients diagnosed between 2001 and 2005 in the Juntendo University Hospital. Remission of hematuria was defined as three consecutive dipstick test results of ( −) to ( ±) or a red blood cell count < 5 in urinary sediment per high-power field during at least 6 months. Remission of proteinuria was defined as three consecutive dipstick results of ( −) to ( ±) during at least 6 months. We categorized four groups according to the remission status which was assessed 2 years after the renal biopsy. The primary outcome was a 50% increase in the serum creatinine over the baseline. We evaluated the slope of eGFR decline (mL/min/1.73 m^2^/year) and a decrease in the eGFR of 30% from baseline eGFR as the secondary outcome, respectively.

**Results:**

A total of 74 patients (male: 47.3%, median age: 30 years) were included and were followed for a median of 86.5 months. During the period, forty-one patients achieved neither remission of proteinuria nor hematuria (NR). Twelve patients met the primary study outcome. A survival analysis revealed that the NR had the worst prognosis and the steepest slope of eGFR decline.

**Conclusion:**

Although further validation in a large cohort is necessary, these novel remission criteria for IgAN patients appear to predict the renal prognosis.

**Supplementary Information:**

The online version contains supplementary material available at 10.1007/s10157-021-02069-w.

## Introduction

Immunoglobulin A nephropathy (IgAN) is the most common form of chronic glomerulonephritis in Japan. In the absence of therapeutic intervention, approximately 40% of IgAN patients progress to end-stage kidney disease (ESKD) within 20 years [1]. Renin–angiotensin–aldosterone system inhibitor (RAS-I) comprises the most common therapeutic approach [2]. However, Pozzi et al. [3] first reported the efficacy of steroid pulse therapy, and more recent studies have increasingly supported these findings. Moreover, Hotta et.al [4] reported the efficacy of tonsillectomy combined with steroid pulse (TSP) therapy as the almost half patients achieved the clinical remission and none of these patients showed progressive deterioration for renal function. This method was quickly adapted after Japanese nephrologists realized its efficacy. Today, TSP therapy is the standard of treatment for Japanese adult IgAN patients [5].

Annual check-ups for urinalysis are well developed in Japan and have facilitated the early diagnosis of IgAN in many cases. Consequently, the administration of immunosuppressive agents to patients with early-stage disease frequently led to the clinical remission of IgAN [5]. Although several studies have defined remission as an outcome [6–8], no uniform definition of this outcome has been determined. In 2013, the special IgA Nephropathy Study Group in Progressive Renal Diseases Research, in affiliation with Research on Intractable Disease by the Ministry of Health, Labor, and Welfare in Japan proposed new remission criteria for IgAN based on a nationwide opinion survey [9]. Although the criteria reflected the opinions of Japanese nephrologists, the clinical impact on renal prognosis remained unclear. Therefore, we investigated the utility of these criteria as a surrogate endpoint in the progression of IgAN in present study.

## Materials and methods

### Study design and participants

This single-center, longitudinal retrospective study included patients with IgAN who were diagnosed by renal biopsy in the Juntendo University Hospital between Jan 2001 and Dec 2005, and were followed for more than 2 years. A diagnosis of IgAN was based on the immunofluorescent detection of glomerular mesangial deposits predominantly comprising IgA and the absence of clinical or laboratory evidence of systemic lupus erythematosus, IgA vasculitis or liver cirrhosis. The study protocol was approved by the ethics committee of the Juntendo University (No.896) and was conducted in accordance with the principles of the Declaration of Helsinki [10].

### Baseline characteristics

Baseline clinical characteristics were collected from the medical records at the time of renal biopsy. The clinical characteristics included age at the time of diagnosis; sex, systolic blood pressure, serum total protein, serum albumin, blood urea nitrogen, and serum creatinine, estimated glomerular filtration rate (eGFR) calculated using the Modification of Diet in Renal Disease equation [11]. Serum IgA, complement C3 and 24-h urinary protein and red blood cell count in urinary sediment were also included. The histological variables were evaluated according to the Oxford Classification [12] [13], i.e., mesangial hypercellularity (M), endocapillary hypercellularity (E), segmental sclerosis (S), tubulointerstitial and atrophy/fibrosis (T), and crescents (C). These histological gradings were confirmed by both nephrologists and renal pathologists.

### Characteristics at follow-up

During the outpatient follow-up period, we collected the serum creatinine, urinary creatinine, urinary protein, and red blood cell count in the urinary sediment from the medical records every 3 months. Therapeutic interventions, including tonsillectomy, immunosuppressant (including corticosteroid) use, anti-platelet agent use, and renin–angiotensin–aldosterone system inhibitor (RAS-I) use for more than 3 months, were recorded at the time of follow-up.

### Definitions of remission and outcomes

For this study, a remission of hematuria was defined as three consecutive dipstick test results of ( −) to ( ±) or a red blood cell count < 5 in urinary sediment per high-power field during at least 6 months. A remission of proteinuria was defined as three consecutive dipstick results of ( −) to ( ±) during at least 6 months [9]. We determined each hematuria remission and proteinuria remission for every patient at 2 years after the renal biopsy. According to this remission status, we categorized the patients into four groups as follows: both proteinuria and hematuria remission (CR), the remission of proteinuria only (UP-R: proteinuria remission and non-remission of hematuria), the remission of hematuria only (UH-R: non-remission of proteinuria and remission of hematuria), both non-remission proteinuria and non-remission hematuria (NR).

The primary study outcome was a 50% increase in the baseline creatinine level. The secondary outcomes were the slope of eGFR decline (mL/min/1.73 m^2^/year) and a decrease in the eGFR of 30% from baseline eGFR, respectively. The slope of eGFR decline was calculated using the principle of the least-squares methods in a linear regression model.

### Statistical analysis

Statistical analysis was performed using Stata Version 14 (Stata Corp, College Station, TX, USA). Normally distributed continuous variables were expressed as means ± standard deviations and were compared using Student’s *t* test. Non-normally distributed continuous variables were expressed as medians with interquartile ranges and were compared using the Mann–Whitney *U* test. Categorical variables are expressed as numbers with percentages and were analyzed using the *χ*^2^ test or Fisher’s exact test. Survival rates were estimated using the Kaplan–Meier method, and differences in survival between groups were determined by the log-rank test. The univariate analysis of the eGFR slope was conducted using the linear regression method and was followed by a multiple regression analysis. All tests were two-sided with *p* < 0.05 were considered statistically significant, and all confidence intervals (CIs) were compared at the 95% level.

## Results

### Baseline characteristics

**Fig. 1:**
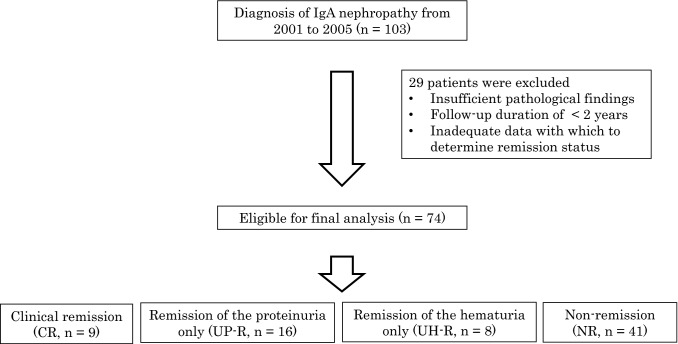
Flow diagram of the study. A total of 103 patients were diagnosed with IgA nephropathy during the study period, of whom 74 were included in the final analysis. Nine, 16, and eight patients achieved clinical remission (CR), remission of proteinuria only (UP-R), or remission of hematuria only (UH-R), respectively. Forty-one patients achieved neither remission of proteinuria nor hematuria (NR)

A total of 103 patients were diagnosed with IgAN during the study period. We excluded 29 patients due to several reasons as follows: patients had insufficient pathological findings, patients were followed up for less than two years, and patients had inadequate data to determine the remission. Therefore, the final analysis included 74 patients (Fig. 1). The characteristics of patients at the time of renal biopsy are presented in Table 1. The patients were followed for a median of 86.5 (range: 71–104) months. The mean eGFR was 87.8 mL/min/1.73 m^2^, and the median urinary protein level was 0.59 (0.21–1.3) g/day. Sixteen patients (21.6%) underwent tonsillectomy with steroid pulse (TSP) therapy, 6 (8.1%) patients underwent the steroid monotherapy, and 4 (5.4%) patients underwent the tonsillectomy only. Thirty-seven patients (50.0%) were prescribed anti-platelet agent, while 32 (43.2%) were prescribed RAS-I.

**Table 1:** Baseline characteristics of the patients and outcomes

	All patients (*n* = 74)	Clinical remission (*n* = 9)	Remission of proteinuria only (*n* = 16)	Remission of hematuria only (*n* = 8)	Non-remission (*n* = 41)	*p*
Age	30 (25–42)	30 (22–37)	29 (24–37)	29 (24–37)	33 (26–48)	0.20
Sex (male %)	35 (47.3%)	4 (44.4)	8 (50.0)	3 (37.5)	20 (57.1)	0.94
SBP (mmHg)	119.1 (± 15.6)	117.0 (± 16.4)	114.2 (± 14.3)	119.5 (± 8.8)	121.3 (± 16.9)	0.47
TP (g/dL)	6.9 (± 0.6)	7.1 (± 0.3)	6.9 (± 0.7)	6.9 (± 0.7)	6.8 (± 0.7)	0.62
Alb (g/dL)	4.1 (± 0.5)	4.3 (± 0.4)	4.3 (± 0.5)	4.2 (± 0.5)	4.0 (± 0.4)	0.05
S-Cre (mg/dL)	0.79 (± 0.26)	0.71 (± 0.16)	0.75 (± 0.32)	0.78 (± 0.19)	0.83 (± 0.26)	0.51
eGFR (ml/min/1.73 m^2^)	87.8 (± 26.8)	94.1 (± 18.1)	99.6 (± 29.6)	94.1 (± 26.5)	80.6 (± 25.9)	0.07
IgA (mg/dL)	312.5 (± 96.0)	348.3 (± 82.2)	273.4 (± 77.1)	264.6 (± 105.6)	329.2 (± 98.1)	0.06
C3 (mg/dL)	102.5 (± 16.5)	109.3 (± 17.6)	92.6 (± 9.6)	102.5 (± 15.6)	104.9 (± 17.5)	0.04
IgA/C3	3.1 (± 0.96)	3.3 (± 0.93)	3.0 (± 0.94)	2.6 (± 1.0)	3.2 (± 0.97)	0.47
UP (g/day)	0.59 (0.21–1.30)	0.21 (0.14–0.28)	0.51 (0–1.2)	0.63 (0.12–1.14)	0.89 (0.37–1.9)	0.02
UOB						
> 30/HPF (*n*, %)	53 (76.8)	7 (77.8)	12 (80.0)	4 (50.0)	30 (81.1)	0.11
21–30/HPF (*n*, %)	2 (2.9)	0 (0.0)	0 (0.0)	1 (12.5)	1 (2.7)	
16–20/HPF (*n*, %)	5 (7.3)	0 (0.0)	2 (13.3)	0 (0.0)	3 (8.1)	
11–15/HPF (*n*, %)	3 (4.4)	1 (11.1)	0 (0.0)	2 (25.0)	0 (0.0)	
6–10/HPF (*n*, %)	3 (4.4)	0 (0.0)	1 (6.7)	0 (0.0)	2 (5.4)	
1–5/HPF (*n*, %)	3 (4.4)	1 (11.1)	0 (0.0)	1 (12.5)	1 (2.7)	
Pathological findings						
M1 (*n*, %)	14 (18.9%)	2 (22.2%)	4 (25.0%)	3 (37.5%)	5 (12.2%)	0.24
E1 (*n*, %)	19 (25.7%)	3 (33.3%)	5 (31.3%)	1 (12.5%)	10 (24.4%)	0.79
S1 (*n*, %)	61 (82.4%)	9 (100%)	13 (81.2%)	5 (62.5%)	34 (82.9%)	0.24
T1 (*n*, %)	40 (54.1%)	4 (44.4%)	5 (31.3%)	5 (62.5%)	26 (63.4%)	0.13
T2 (*n*, %)	3 (4.1%)	0 (0%)	1 (5.9%)	0 (0%)	2 (4.9%)	1.00
C1 (*n*, %)	40 (54.1%)	4 (44.4%)	11 (68.8%)	3 (37.5%)	22 (53.4%)	0.46
Follow-up months	86.5 (71–104)	94 (88–100)	82 (72–93)	91 (114–127)	83.0 (69–111)	0.70
Treatment options						
Tonsillectomy with steroid pulse therapy (*n*, %)	16 (21.6%)	2 (22.2%)	3 (18.8%)	3 (37.5%)	8 (19.5%)	0.89
Steroid pulse monothrapy (*n*, %)	6 (8.1%)	0 (0%)	1 (6.3%)	3 (37.5%)	2 (4.9%)	0.04
Tonsillectomy only (*n*, %)	4 (5.4%)	1 (11.1%)	0 (0%)	0 (0%)	3 (7.3%)	0.48
Anti-platelet agent (*n*, %)	37 (50.0%)	3 (33.3%)	8 (50.0%)	6 (75.0%)	20 (48.8%)	0.53
RAS-I (*n*, %)	32 (43.2%)	1 (11.1%)	4 (25.0%)	6 (75.0%)	21 (51.2%)	< 0.001
50% increase in serum creatinine from baseline (*n*, %)	12 (16.2%)	0 (0%)	1 (6.3%)	0 (0%)	11 (26.8%)	0.07
30% decrease in eGFR from baseline (*n*, %)	18 (24.0%)	0 (0%)	2 (2.7%)	3 (4.1%)	13 (17.6%)	0.09
Slope of decline in eGFR (ml/min/1.73 m^2^/year)	− 1.74 (± 2.56)	− 1.16 (± 1.9)	− 0.53 (± 2.2)	− 2.10 (± 1.9)	− 2.27 (± 2.8)	0.04

### Remission status

Twenty-five patients (33.8%) achieved remission of proteinuria, yielding an incidence of 4.8 per 100 person-years. Seventeen patients (23.0%) achieved remission of hematuria, yielding an incidence of 3.3 per 100 person-years. Nine patients (12.2%) achieved a CR, with an incidence rate of 1.7 per 100 person-years. Consequently, we categorized the patients as follows: CR, 9 patients; UP-R, 16 patients; UH-R, 8 patients; and NR, 41 patients. At the time of renal biopsy, the urinary protein level differed significantly between the four groups (*p* = 0.02). Of the patients who were underwent TSP therapy, 2 patients achieved CR, 3 patients achieved UP-R, 3 patients achieved UH-R, and 8 patients achieved neither remission of proteinuria nor remission of hematuria (SupplementTable 1).

### Primary outcome: 50% increase in baseline creatinine level

**Fig. 2:**
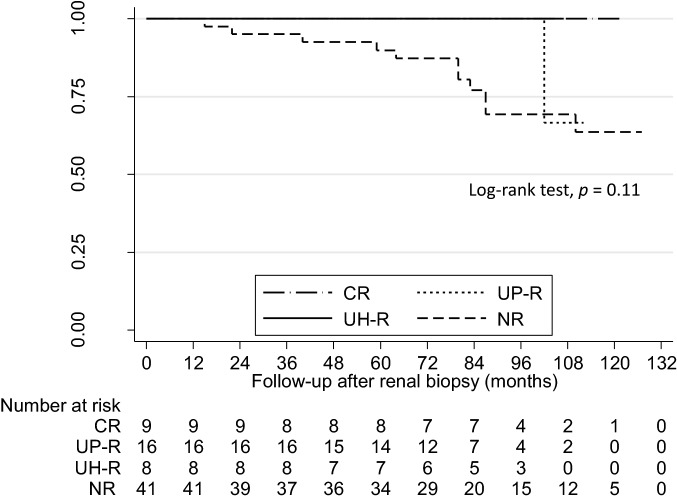
Cumulative renal survival rates according to remission status. Abbreviations: *CR* Clinical remission, *UP-R* Remission of proteinuria only, *UH-R* Remission of hematuria only, *NR* non-remission

Twelve patients (16.2%) developed a 50% increase in the serum creatinine level during the observation period, including 11 patients in the NR. This outcome was not observed in the UH-R and CR groups. A Kaplan–Meier analysis (Fig. 2) demonstrates a lower cumulative renal survival rate in the NR than that in the other groups (log-rank test, *p* = 0.11). Two patients who were underwent TSP therapy developed a 50% increase the serum creatinine, and all of them categorized NR (Supplement Table 1).

### The slope of eGFR decline and a decrease in the eGFR of 30% from baseline

As shown in Table 1, the four groups differed significantly with respect to the mean slope of the decline in eGFR (*p* = 0.02). The slope of the decline was the steepest among the four groups in the NR group and the lowest in the UP-R group. Tables 2 and 3 present the associations between the slope of the decline in eGFR and the covariates in the regression model. Notably, UP-R was the only parameter that exhibited a significant association with the decline in eGFR (ß coefficient, 1.61; 95% CI, 0.22–3.00; *p* = 0.02) in a multivariate model adjusted for age, systolic blood pressure, total protein, eGFR at baseline, and urinary protein at baseline. In the patients who underwent the TSP therapy, the mean slope of the decline in eGFR showed that the same tendency (Supplement Table 1). A decrease in the eGFR of 30% from baseline was observed in 18 patients (24.0%) including 13 patients in the NR. These results tended to be similar to 50% increase in baseline creatinine level.

**Table 2:** Univariate analysis of the associations of clinical factors with the slope of the decline in the estimated glomerular filtration rate (eGFR) per year

Factor	*β* coefficient	95% CI	*p*
Sex (male)	0.13	− 1.07 to 1.33	0.83
Age	− 0.01	− 0.06 to 0.03	0.55
SBP (mmHg)	0.01	− 0.03 to 0.05	0.64
S-Cre (mg/dL)	− 0.65	− 2.95 to 1.65	0.58
eGFR at baseline	− 0.01	− 0.03 to 0.02	0.66
TP (g/dL)	0.95	− 0.02 to 1.88	0.05
Alb (g/dL)	1.1	− 0.20 to 2.4	0.10
UP (g/day)	− 0.53	− 0.99 to − 0.05	0.03
UOB (per degree)	0.08	− 0.33 to 0.48	0.71
Pathological findings			
M1	0.63	− 0.89 to 2.15	0.41
E1	− 0.02	− 0.16 to 0.13	0.83
S1	− 0.71	− 2.28 to 0.85	0.37
T1	0.02	− 1.18 to 1.22	0.98
T2	− 0.31	− 3.34 to 2.72	0.84
C1	− 0.17	− 1.37 to 1.03	0.77
Non-remission (NR)	(Reference)	–	–
Remission of the hematuria only (UH-R)	− 0.40	− 2.33 to 1.52	0.68
Remission of the proteinuria only (UP-R)	1.54	0.13 to 2.94	0.03
Clinical remission (CR)	0.66	− 1.16 to 2.48	0.47

**Table 3:** Multiple regression analysis of the association between the remission status and the slope of the decline in the estimated glomerular filtration rate (eGFR) per year

Remission status	*β* coefficient ^a^	95% CI	*p*
Non-remission (NR)	(Reference)	–	–
Remission of the hematuria only (UH-R)	− 0.48	− 2.37 to 1.41	0.62
Remission of the proteinuria only (UP-R)	1.61	0.22 to 3.00	0.02
Clinical remission (CR)	0.13	− 1.68 to 1.93	0.89

## Discussion

In present study, 74 patients with IgAN were followed for a median of 86.5 months, during which 33 patients achieved at least partial remission and 41 patients did not achieve remission. Moreover, 12 patients met the primary outcome of a 50% increase in the baseline creatinine level, of whom 11 patients had failed to achieve both hematuria and proteinuria remission. The slope of the decline in eGFR was the steepest among patients who in NR. Several previous studies have used remission criteria as an outcome, however, to the best of our knowledge, our study was the first to investigate the association of clinical remission and renal prognosis in Japanese adult IgAN patients.

In Japan, school-aged children and adults undergo annual routine screenings for urinary abnormalities [14, 15]. Accordingly, Japanese nephrologists can provide early-stage immunotherapeutic interventions (including tonsillectomy), and affected patients frequently achieve remission of IgAN. However, the definition of remission varies among Japanese nephrologists due to the lack of uniform criteria. In 2013, Suzuki et al. [9] proposed novel criteria based on the results of a consciousness survey of Japanese nephrologists. In the present study, we observed that the incidence of a 50% increase in the baseline creatinine level was significantly higher (11 patients, 14.9%) and the slope of the decline in eGFR was significantly steeper in the NR than in other groups (CR, UP-R, and UH-R) (Table 1). These results suggest that the inability to achieve remission within 2 years after the renal biopsy is a prognostic factor for renal deterioration and demonstrate that the remission criteria proposed in 2013 are reflective of renal prognosis.

Tonsillectomy may affect the upstream pathway of the pathogenic mechanism by eliminating antigenic stimuli from the tonsillar mucosa, and steroid pulse therapy may reduce responsible B cells clones which are disseminated to bone marrow from mucosa and ameliorate local inflammation in kidney [16]. According to this hypothesis, Hotta et al. [4] suggested that the TSP therapy may improve the long-term prognosis for renal function. In this study, 16 patients were received TSP therapy, and half of the patients had some of the remission status (CR: 2 patients, UP-R: 3 patients, UH-R: 3 patients,). Of those, none of the patients had some of the remission status obtained the primary outcome, and the slope of the decline in eGFR in NR was steepest. Although further studies are needed due to the small number of cases in this study, our result supports that the non-remission of IgAN is an indicator of progression of renal insufficiency in patients who were underwent TSP therapy.

Recently, a decrease in the eGFR of 30% from the baseline has been proposed as a surrogate marker for chronic kidney disease [17]. We evaluated that the eGFR of 30% from baseline as the secondary outcome, and showed that the similar results as when using the primary outcome. On the other hand, the decrease in the eGFR of 30% from baseline occurred only two patients within two years after the renal biopsy. This outcome may not accurately discriminate IgAN patients in early stage, thus predictive value of the remission criteria based on the urinary findings should be considered.

In this study, patients who achieved remission of proteinuria during the 2 years after the renal biopsy had a significantly better prognosis than those who failed to achieve remission (Table 1). Moreover, UP-R was found to associate significantly with the slope of the decline in eGFR in both the univariate and multivariate models (Tables 2 and 3). Proteinuria is a well-known prognostic factor for IgAN. Reich et al. [7] reported that both proteinuria at the time of renal biopsy and the mean amount of proteinuria during follow-up were prognostic factors in this patient population. Besides, the average albuminuria over time was reported as a prognostic factor in these days [18, 19]. Our findings confirm the status of proteinuria remission during the early stage of disease as a significant renal prognostic factor. Moreover, our findings suggest that strong immunosuppressive treatment, which drives remission, should be considered for patients with early-stage IgAN.

Hematuria is one of the characteristic symptoms of IgAN. Also, microscopic hematuria has been identified as a strong risk factor for progression to ESKD [20, 21]. Consequently, the disappearance of hematuria after the treatment with corticosteroids and immunosuppressants has been identified in patients with progressive IgAN [22]. However, several other studies have not identified an association of the degree of hematuria with pathological lesions [23] or with negative outcomes [24], and the prognostic value of this factor remains controversial in the Japanese patients. Previous authors proposed a multi-hit hypothesis regarding the pathogenesis of IgAN [25] that highlights the importance of galactose-deficient IgA1 (Hit 1), IgG or IgA autoantibodies that recognize Gd-IgA1 (Hit 2), the subsequent immune complexes formation (Hit 3), and glomerular deposition of immune complexes (Hit 4). Suzuki et al. [26] further clarified the association between the degree of hematuria and that of galactose-deficient IgA1 (Hit 1), while Sevillano et al. [27] demonstrated that the average duration of hematuria during follow-up had a significant influence on the progression of IgAN. In this study, no patients who achieved remission of hematuria met the primary outcome of a 50% increase in creatinine over the baseline (Table 1). It is suggested that hematuria may play a key role in the pathogenesis of IgAN and may indicate a disease activity of IgAN. Therefore, hematuria remission could be useful as a treatment indicator.

This study had several limitations. First, this study was the small number of patients. Further large cohort study needed to evaluate our findings. Second, the primary study outcome was observed in only 12 patients, which was lower than the rates reported by other cohort studies in Japan. Therefore, individual outcome may have been overestimated. Third, we evaluated the remission at 2 years after the diagnostic renal biopsy, during which period, nearly all study patients had received the initial therapy. However, we were unable to evaluate the long-term efficacy of remission of hematuria and proteinuria. Fourth, patients who were available for long-term follow-up had relatively good adherence for the treatment, and, therefore, we cannot exclude the potential for survival bias.

## Conclusion

Present findings indicate that the failure to achieve a remission of IgAN is associated with deterioration of renal function. Although further validation with a large cohort study is necessary, the recently proposed remission criteria for IgAN could predict renal prognosis in this population.

## Supplementary Information

Below is the link to the electronic supplementary material.

Supplementary file1 (XLSX 16 kb)
